# Prediction of gross calorific value from coal analysis using decision tree-based bagging and boosting techniques

**DOI:** 10.1016/j.heliyon.2023.e23395

**Published:** 2023-12-07

**Authors:** Tanveer Alam Munshi, Labiba Nusrat Jahan, M. Farhad Howladar, Mahamudul Hashan

**Affiliations:** Department of Petroleum and Mining Engineering, Shahjalal University of Science and Technology, Sylhet, 3114, Bangladesh

**Keywords:** Gross calorific value, Tree-based ensemble techniques, Bagging models, Boosting models, Coal, Proximate and ultimate analyses

## Abstract

The calorific value of any fuel is one of the crucial parameters to grade fuel's burning capability. The bomb calorimeter has historically been used to calculate coal's gross calorific value (GCV). However, for many years, engineers and scientists were trying to measure coal's GCV without a bomb calorimeter, using only laboratory-derived ultimate and/or proximate analyses to eliminate tedious and time-consuming laboratory analyses. In this study, Extra trees, Bagging, Decision tree, and Adaptive boosting are developed for the first time in coal's GCV modeling. In addition, the prediction and computational efficiency of previously applied decision tree-based algorithms, such as Random forest, Gradient boosting, and XGBoost are investigated. Well-established empirical models, namely Schuster, Mazumdar, Channiwala and Parikh, Parikh et al. and Central Fuel Research Institute of India are examined to compare their efficiency with newly developed algorithms. Proximate and ultimate analysis parameters are ranked based on their significance in GCV modeling. The studied models are tuned using an exhaustive grid search technique. Statistical indexes, such as explained variance (EV), mean absolute error (MAE), coefficient of determinant (R^2^), mean squared error (MSE), maximum error, minimum error, and mean absolute percentage error (MAPE) are used to critique these models. To accomplish the goals, 7430 data points containing ten coal features, such as ash, moisture, fixed carbon, volatile matter, hydrogen, carbon, sulfur, nitrogen, oxygen, and GCV are selected from the U.S. Geological Survey Coal Quality (COALQUAL) database. It has been found that, due to simplicity and location-specific constraints, empirical models could not correlate proximate and/or ultimate analyses with GCV. Bagging and boosting techniques tested here performed well with the coefficient of determinant ($R^{2}$) of over 0.97. The XGBoost model outperforms other tree-based algorithms with the most significant coefficient of determinant ($R^{2}$ of 0.9974) and lowest error values (MSE of 14703.3, max_error of 1027.2, MAE of 89.2, MAPE of 0.009). The studied models' ranking (highest to lowest) based on their performance are XGBoost, Extra trees, Random forest, Bagging, Gradient boosting, Decision tree, and Adaptive boosting. The correlation heatmap and scatterplots used here clearly indicate that oxygen and carbon are the utmost significant, whereas volatile matter and sulfur are the least essential rank parameters for GCV modeling. The strategy suggested in this research can aid engineers/operators in obtaining a rapid and accurate determination of the GCV with a few coal features, thus lessening complicated, tedious, expensive, and time-consuming laboratory efforts.

## Highlights

• Extra trees, Bagging, Decision tree, and Adaptive boosting are developed for the first time in coal's GCV modeling.
• Proximate and ultimate analysis parameters are ranked based on their significance in GCV modeling.
• The XGBoost is superior to any previously applied model with a coefficient of determinant of 0.9975.
• Well-established empirical relationships are inappropriate for GCV modeling.
• Oxygen ($C_{O}$) and Carbon ($C_{C}$) contents are the most significant rank parameters for GCV modeling.

## 1 Introduction

Coal is an incredibly heterogeneous natural material encountered on Earth. It may involve up to 76 of the 98 naturally developing chemical components, most appearing as traces [1]. Assessments, such as calorific value, ultimate analysis, and proximate analysis determine the energy quality of coal. The weight percentages of ash ($C_{A}$), moisture ($C_{M}$), fixed carbon ($F_{C}$), and volatile matter ($V_{m}$) are measured by proximate analysis [2]. These four components involve 100 % of all chemical constituents of coal. The elements reported in an ultimate analysis are hydrogen ($C_{H}$), carbon ($C_{C}$), sulfur ($C_{S}$), nitrogen ($C_{N}$), and oxygen ($C_{O}$) [3,4]. These five components together determine the quantity of air necessary for the complete combustion of coal [5]. The other usual laboratory assessment is the gross calorific value (GCV), a fundamental property when coal is planned to be employed as fuel. GCV is coal's most influential rank property and depends on the composition of minerals and maceral [6]. The most exact measurement is GCV generated from a coal sample in a laboratory using an adiabatic bomb calorimeter, but the process is expensive, time-consuming, and tedious [4,7]. These limitations forced the formulation of different empirical relationships to estimate GCV using the ultimate and/or proximate analyses. Table 1 lists the most widely used empirical models to estimate the GCV from ultimate and/or proximate analyses. These linear algebraic correlations have several advantages, such as: (i) they are simple and fast methods for valuing the GCV, thus saving the cost and labor required in its laboratory measurement, (ii) they can be used to model the performance of coal-based combustion, pyrolysis, and gasification processes, and (iii) in order to investigate how the ultimate and proximate analyses of a coal affect process performance, they give algebraic equations linking GCV with the ultimate and/or proximate analyses [7]. However, the prime disadvantage of these non-universal algebraic formulations is their poor accuracy in estimating GCV from proximate and/or ultimate analyses [8].

**Table 1:** Most widely used empirical models applied to estimate the GCV from proximate and/or ultimate analyses.

Reference	Input feature (s)	Target (s)	Used data	Equations
Goutal [9]	$F_{C}$, $V_{m}$	GCV	Proximate data	$Q=4.183\times 10^{-3}\times \left(82\times F_{C}+K\times V_{m}\right)\;\left(\text{MJ}/\text{kg}\right)$
Schuster [10]	$V_{m}$	GCV	Proximate data	$Q=4.183\times 10^{-3}\left(8000+V_{m}\times \left(70-1.65\times V_{m}\right)\right)\;\left(\text{MJ}/\text{kg}\right)$
Spooner [11]	$C_{O},V_{m}$	GCV	Ultimate and proximate data	$Q=4.183\times 10^{-3}\times \left(8781+{19\times V}_{m}-144\times C_{O}\right)\;\left(\text{MJ}/\text{kg}\right)$
Mazumdar [12]	$C_{M},V_{m}$	GCV	Proximate data	$Q=4.184\times 10^{-3}\times \left(9170-{16\times V}_{m}-60\times C_{M}\times \left(1-0.001\times C_{M}\right)\right)\;\left(\text{MJ}/\text{kg}\right)$
Mazumdar [13]	$C_{O},C_{H},C_{C},C_{A}$	GCV	Ultimate and proximate data	$Q=13.03\times C_{O}+\left(100-C_{\text{MM}}\right)\times \left(\frac{0.238\times C_{H}}{C_{C}-0.0065}\right)-0.007\times C_{A}\;\left(\text{MJ}/\text{kg}\right)$
CFRII formulae	$C_{A},C_{M}$	GCV	Proximate data	$\text{UHV}=4.184\times \left(8900-138\times \left(C_{A}+C_{M}\right)\right)\;\left(\text{MJ}/\text{kg}\right)$
Channiwala & Parikh [14]	$C_{C},C_{H},C_{S},C_{O},C_{N},C_{S}$	GCV	Ultimate and proximate data	$Q=0.3491\times C_{C}+1.1783\times C_{H}+0.1055\times C_{S}-0.1034\times C_{O}-0.0151\times C_{N}-0.0211\times C_{A}\;\left(\text{MJ}/\text{kg}\right)$
Parikh et al. [15]	$F_{C},C_{A},V_{m}$	GCV	Proximate data	$Q=0.3536\times F_{C}+0.1559\times V_{m}-0.0078\times C_{A}\;\left(\text{MJ}/\text{kg}\right)$

Computer-based soft computing methods, such as support vector machine (SVM), particle swarm optimization (PSO), fuzzy logic (FL), fuzzy decision tree (FDT), artificial neural networks (ANN), wavelet neural network (WNN), genetic algorithm (GA), adaptive neuro-fuzzy inference system (ANFIS), co-active neuro-fuzzy inference system (CANFIS), convolutional neural network (CNN), imperialist competitive algorithm (ICA) and, recurrent neural network (RNN) have recently been advanced in research areas of scientific, engineering, technological, and industrial courses [[16], [17], [18], [19], [20], [21], [22], [23]]. These state-of-the-art mathematical modeling tools can capture high dimensional complex data, recognize inherent highly complex links from input-output data, find optimum patterns, and forecast target parameters [24].

A little research has been conducted over the past few decades to determine the applicability of data-driven modeling in forecasting GCV from proximate and/or ultimate analyses (Table 2). Several multiple variable regression (MVR) models have been prepared to increase the accuracy of GCV prediction [4,24,25]. The application of smart tree-based intelligent methods is limited in GCV prediction. Tree-based intelligent methods, namely regression tree, random forest, gradient boosting tree, and XGBoost were applied by Bui et al. [8], Matin and Chelgani [26], Ahmed et al. [27], and Chelgani [28], respectively (Table 2). They applied these techniques to different datasets with different sample sizes. However, wide-ranging fine-tuning is absent in these studies.

**Table 2:** Studies conducted to predict GCV from proximate and/or ultimate analyses using soft computing and intelligent statistical modeling.

Year	Reference	Input feature (s)	Target (s)	Algorithm (s)	Testing score (s)	Statistical metrics	Sample size	Input features ranking	Comments/remarks
2007	Patel et al. [29]	C_M_, V_M_, C_A_ C_C_, C_H_, FC, C_S_, C_N_, C_O_	GCV	ANN	RMSE: 0.514 MJ/kg (best) R^2^: 0.997 (best)	R^2^, RMSE, APE	79	Yes	1. ANNs are accurate GCV predictors and better than conventional mathematical correlations. 2. CO, CC, and CA are the most important input variables for GCV prediction.
2009	Mesroghli et al. [30]	C_M_, V_M_, C_A_ C_C_, C_H_, FC, C_S_, C_N_, C_O_	GCV	ANN MVR	R^2^: 0.947 R^2^: 0.985	R^2^	4540	No	1. Three-layer feed-forward error back propagation technique is used.
2010	Chelgani et al. [31]	C_S_, $V_{M}$, C_O_, C_H_, C_C_	R_max_, GCV	ANN MVR	R^2^: 0.84 (GCV) R^2^: 0.69 (GCV)	R^2^	1018	No	1. ANN gave more accurate results compared to MVR.
									2. Rmax and GCV are predicted simultaneously.
2015	Feng et al. [32]	C_M_, V_M_, C_A_	GCV	ANN	R^2^: 0.876 MSE: 0.956 [MJ/kg]^2^	R^2^, MSE, MAPE, MAE	76	No	1. All of the techniques were capable of trackling variation in GCV.
				SVM	R^2^: 0.945 MSE: 0.423 [MJ/kg]^2^				2. SVM gave better performance in terms of generalization error.
				ACE	R^2^: 0.912 MSE: 0.673 [MJ/kg]^2^				
2016	Matin and Chelgani [26]	C_M_, V_M_, C_A_, C_C_, C_H_, C_S_, C_N_	GCV	RF	R^2^: 0.97 (Proximate) R^2^: 0.99 (Ultimate)	R^2^, SD Mean residual	6339	No	1. RF proved to be a powerful model for feature rankings.
				MVR	R^2^: 0.97 (Proximate) R^2^: 0.99 (Ultimate)				2. Both MVR and RF gave reasonable performance in predicting GCV.
2017	Hadavandi et al. [33]	C_M_, $V_{M}$, C_A_, C_C_, C_H_, C_N_, Rmax, Inersts, Vitrinites, Lipnites, Organic sulfur, Pyrite sulfur, Sulfate sulfur	GCV	SVR	R^2^: 0.98	R^2^	924	Yes	1. SVR is used for both GCV prediction and variable importance measurement. 2. Apart from proximate and ultimate analyses, other properties are considered as input features. 3. Two SVR models were developed. One took all input features and the other took top four important input features.
2017	Lu et al. [34]	C_C_, C_H_, C_O_, Si, Al, Fe, Ca, Mg, K, Na, and Ti	GCV	GA-ANN	RMSE: 0.27 (MJ/kg) MAE: 0.39 (MJ/kg)	R^2^, RMSE MAE, MSD	27	No	1. GA-based ANN is developed to obtain rapid online measurement of GCV. 2. GA-ANN can provide quick prediction of GCV with high accuracy.
2017	Wen et al. [35]	C_M_, V_M_, C_A_ C_C_, C_H_, FC, C_S_, C_N_, C_O_	GCV	MWNN GWNN	MAE: 0.17 (MJ/kg) SD: 0.07 MAE: 0.20 (MJ/kg) SD: 0.05	R^2^, MAE SD	6556	No	1. WNN provides similar outcomes as other methods like MVR, ANN, GA, and multivariate nonlinear regression.
2018	Açikkar and Sivrikaya [25]	C_M_, V_M_, C_A_, FC	GCV	MLR MLP GRNN RBFNN	R^2^: 0.973 (best) R^2^: 0.984 (best) R^2^: 0.984 (best) R^2^: 0.986 (best)	R^2^, RMSE, MAE, MAPE	6520	Yes	1. RBFNN-based model performed better compared to MLR, GRNN, and MLP. 2. Among all the methods, Moisture and Ash had more positive effect with GCV.
2019	Bui et al. [8]	C_M_, V_M_, C_A_	GCV	SVR MLR CART	R^2^: 0.952 (best) RMSE: 212.9 (Kcal/kg) R^2^: 0.946 RMSE: 225.9 (Kcal/kg) R^2^: 0.946 RMSE: 226.4 (Kcal/kg)	R^2^, RMSE	2327	No	1. RBF kernel turned out to be the kernel which is best suited for PSO-SVR in predicting GCV. 2. PSO-SVR-RBF performed better compared to, PSO-SVR-P, PSO-SVR-L, MLR, and CART.
2019	Wood [36]	C_M_, V_M_, C_A_, C_C_, C_H_, C_S_, C_N_, F_C_, C_O_	GCV	TOB	R^2^: >0.99 RMSE: ≤ 0.3 MJ/kg	R^2^, MSE, RMSE	6339	No	1. TOB is optimized using memetic firefly optimizer, generalized reduced gradient, and evolutionary algorithm.
2020	Ahmed et al. [27]	C_M_, V_M_, C_A_, FC	CCV	BPNN GBT MLR	R^2^: 0.89 R^2^: 0.91 R^2^: 0.80	R^2^	8039	Yes	1. GBT performed better than BPNN, and MLR. 2. GBT is used for feature importance.
2020	Akkaya and Çetin [37]	C_M_, V_M_, C_A_, FC	GCV	GMDH-type NN	R^2^: 0.95	ME, MAE, MAPE, RMSE, R^2^	8501	No	1. Different literatures have been compared with the GMDH-type NN.
2021	Chelgani [28]	C_M_, V_M_, C_A_, C_C_, C_H_, C_S_, C_N_	GCV	SHAP XGBoost Stepwise	R^2^: 0.996 R^2^: 0.994	R^2^, Max difference, Std. error	50	Yes	1. SHAP analysis is performed to interconnect input and output features and to rank input features.
2021	Nguyen et al. [38]	C_M_, V_M_, C_A_	GCV	ANN PSO-ANN	R^2^: 0.962 RMSE: 187.4 (Kcal/kg) R^2^: 0.964 RMSE: 182.4 (Kcal/kg)	RMSE, MAPE, R^2^, VAF	2583	No	1. Although PSO algorithm was successful in optimizing ANN, it didn't give major boost in terms of performance.

Bagging and boosting are helpful techniques for improving any regressor or classifier. The bagging mechanism enables a predictor to be more robust and balanced. It generalizes any predictor exceptionally well in the testing dataset. Additionally, bagging can address overfitting issues [39]. On the other hand, Boosting can convert a mediocre algorithm to a strong learner via weight transfer in an iterative process. XGBoost made boosting technique more popular by achieving computational efficiency in large-scale datasets and via other optimization techniques, namely sparsity-aware split finding algorithm, weighted quantile sketch, and cache-aware block structure [40]. There is an increasing body of literature on modeling complex input-output relationships using bagging and boosting techniques [[41], [42], [43], [44], [45], [46], [47]]. However, comprehensive examination and fine-tuning of Extra trees, Bagging, Decision tree, and Adaptive boosting are absent in GCV prediction from coal analysis.

The above research review recommends that more trustworthy soft computing methods are still desired to increase/improve the estimation accuracy in GCV modeling. The present investigation is conducted to fill in the research gap by achieving several novelties with methodical accuracy, and can be registered as follows:

• Intelligent statistical models, namely bagging, extra trees, and adaptive boosting are developed for the very first time in the field of coal's GCV modeling.
• A thorough comparison of the studied empirical and intelligent tree-based models is used as an illustration of prediction and computing efficiency.
• The ultimate and proximate analyses parameters are ranked based on their importance in GCV modeling.

The rest of the document is segmented into subsequent sections: In Section 2, the foundations of numerous empirical and smart tree-based techniques used in this study are critically addressed. Along with a summary of the tools and procedures used here, Section 2 also includes information on data collection and preprocessing. Section 3 presents findings and discussions based on critical information. A summary of the conclusions and suggestions is provided in Section 4.

## 2 Theory and research methodology

### 2.1 Data collection and treatment

**Fig. 1:**
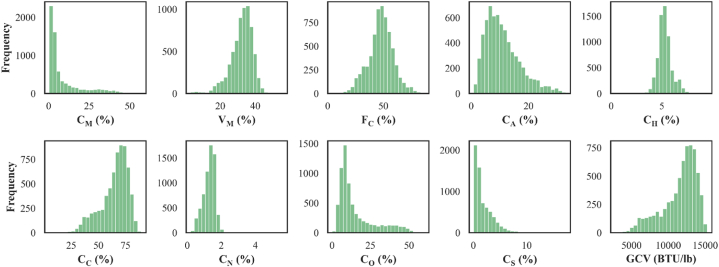
Histogram of GCV, proximate, and ultimate analyses constituents.

The nine proximate and ultimate analyses data consist of $C_{M}$, $V_{m}$, $F_{C}$, $C_{A}$, $C_{H}$, $C_{C}$, $C_{N}$, $C_{O}$, and $C_{S}$ from U.S. Geological Survey Coal Quality (COALQUAL) database, open file report 97–134 [48] are the input variables for this research. Initially, 7430 data points are considered to examine the effectiveness of the suggested strategies in predicting the output variable GCV. Sampling techniques and laboratory experiments conducted to obtain GCV, proximate analysis, and ultimate analysis data are available at http://energy.er.usgs.gov/products/databases/CoalQual/index.htm website. The analysis results presented in the database are on as received basis. In the data preprocessing stage, 848 samples having zero values in either one or more features are removed from primarily considered 7430 data points. The rest 6582 samples are applied to meet the research objectives. Fig. 1 and Table 3 present the results of the statistical analysis of the data samples.

**Table 3:** Statistical analysis on input and output data (as received basis).

Parameters	Sample size	Mean	Median	Standard deviation	Minimum	Maximum	Kurtosis	Skewness
C_M_ (%)	6582	8.76	3.6	10.62	0.4	57.2	2.452	1.83
V_M_ (%)	6582	32.19	33.1	6.26	3	55.7	1.760	−0.99
F_C_ (%)	6582	48.02	48.4	11.23	4.1	87	0.374	−0.07
C_A_ (%)	6582	11.02	9.8	6.01	0.9	32.9	0.609	0.95
C_H_ (%)	6582	5.30	5.28	0.71	0.4	9.5	3.084	0.04
C_C_ (%)	6582	65.05	68.02	12.51	4.65	89.6	0.241	−0.89
C_N_ (%)	6582	1.28	1.3	0.34	0.2	5.6	5.042	0.09
C_O_ (%)	6582	15.45	10.3	11.95	0.2	59.9	0.960	1.41
C_S_ (%)	6582	1.90	1.2	1.73	0.07	17.3	5.289	1.87
GCV (BTU/lb)	6582	11544.41	12204.5	2347.19	2810	15247	0.258	−1.00

**Fig. 2:**
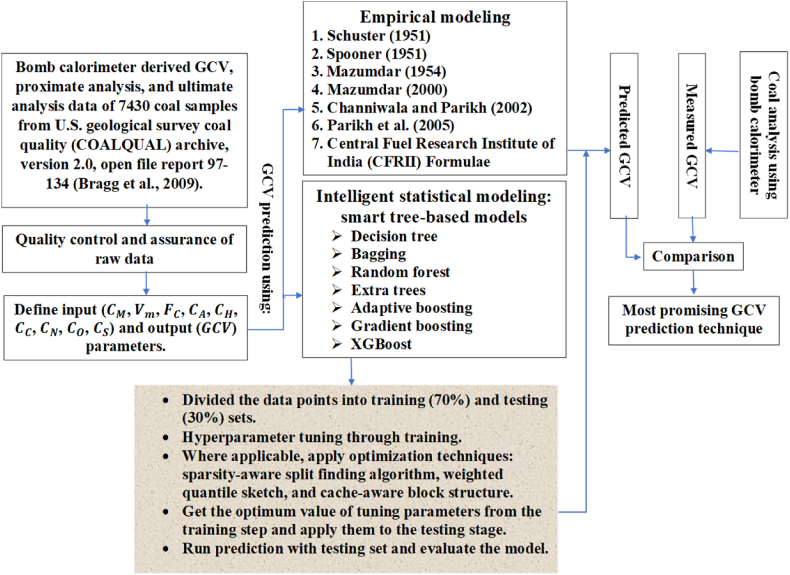
Major steps involved in this research to find an efficient GCV prediction approach.

The proposed empirical and smart tree-based modeling is performed in Python environments. For smart tree-based modeling, python's package scikit-learn is utilized. The major steps of this research are shown in Fig. 2.

### 2.2 Statistical indexes

The performance of the investigated models is evaluated using different metrics, i.e., explained variance (EV), coefficient of determinant (R^2^), mean squared error (MSE), maximum error (max_error), minimum error ((min_error)), mean absolute percentage error (MAPE), and mean absolute error (MAE) The definitions of these performance indicators are as follows (Equations (1), (2), (3), (4), (5), (6), (7))):

(1) $$R^{2}\left(y,\hat{y}\right)=1-\frac{\sum_{i=1}^{n}{\left(y_{i}-\hat{y_{i}}\right)}^{2}}{\sum_{i=1}^{n}{\left(y_{i}-\bar{y}\right)}^{2}}=\frac{{SS}_{res}}{{SS}_{tot}}$$

(2) $$MSE\left(y,\hat{y}\right)=\frac{1}{n_{samples}}\sum_{i=0}^{n_{samples}-1}{\left(y_{i}-\hat{y_{i}}\right)}^{2}$$

(3) $$EV\left(y,\hat{y}\right)=1-\frac{Var\left\{y-\hat{y}\right\}}{Var\left\{y\right\}}$$

(4) $$\max\;\_error\left(y,\hat{y}\right)=\max\;\left(\left|y_{i}-{\hat{y}}_{i}\right|\right)$$

(5) $$\min\;\_error\left(y,\hat{y}\right)=\min\;\left(\left|y_{i}-{\hat{y}}_{i}\right|\right)$$

(6) $$MAE\left(y,\hat{y}\right)=\frac{1}{n_{samples}}\sum_{i=0}^{n_{samples}-1}\left|y_{i}-\hat{y_{i}}\right|$$

(7) $$MAPE\left(y,\hat{y}\right)=\frac{1}{n_{samples}}\sum_{i=0}^{n_{samples}-1}\frac{\left|y_{i}-{\hat{y}}_{i}\right|}{\max\;\left(\epsilon ,\left|y_{i}\right|\right)}$$

Where $\hat{y_{i}}$ refers to the predicted value of the i-th sample; $y_{i}$ denotes the corresponding actual value of the i-th sample, $\hat{y}$ stands for the estimated target output, y represents the corresponding (correct) target output, and ϵ is an arbitrary small but positive number to prevent outcomes that are undefined if y is zero.

### 2.3 Empirical modeling for GCV estimation

Two types of empirical correlations are on hand for coal's GCV evaluation. The first type of relationship is solely dedicated to coal; the other type deals with various fuels, including solid, liquid, and gaseous ones. Goutal [9] was the first researcher to propose a coal-specific empirical relationship associating GCV with $F_{C}$ and $V_{m}$. Schuster [10] correlated the GCV with only $V_{m}$, while Spooner [11] linked the GCV with $V_{m}$ and $C_{O}$ of coal. Mazumdar [12] developed a relationship among GCV, $V_{m}$, and $C_{M}$. Mazumdar [13] recently formulated a more rigorous empirical correlation where he presented that the mineral matter of coal and percentages of $C_{C}$, $C_{H}$, and theoretical $C_{O}$ requirement for the complete coal combustion are needed to estimate GCV. Central Fuel Research Institute of India (CFRII) proposed a correlation among useful heating value (UHV), $C_{A}$, and $C_{M}$. Chaudhury and Biswas (2002) modified the CFRII formulae since the grading and pricing of coal functioned globally rely on GCV, not UHV. The modified version of CFRII model incorporates two new constants (a and b) whose values differ based on the geographical location of the coal. Based on an extensive review conducted on the empirical correlations available for the estimation of GCV of different types of fuels, Channiwala and Parikh [14] developed an algebraic relationship showing that the GCV is associated with the content of $C_{C}$, $C_{H}$, $C_{S}$, and $C_{N}$. The most recent relationship was formulated by Parikh et al. [15] linking GCV with $F_{C}$, $V_{m}$, and $C_{A}$.

The widely used seven empirical correlations, namely Schuster [10], Spooner [11], Mazumdar [12], Mazumdar [13], Channiwala and Parikh [14], Parikh et al. [15], and CFRII Formulae presented in Table 1 are utilized in this study to calculate coal's GCV. After calculating Q (GCV) from the empirical equations, the unit of Q is converted to BTU/lb from Mj/kg.

### 2.4 Smart tree-based modeling for GCV prediction

**Fig. 3:**
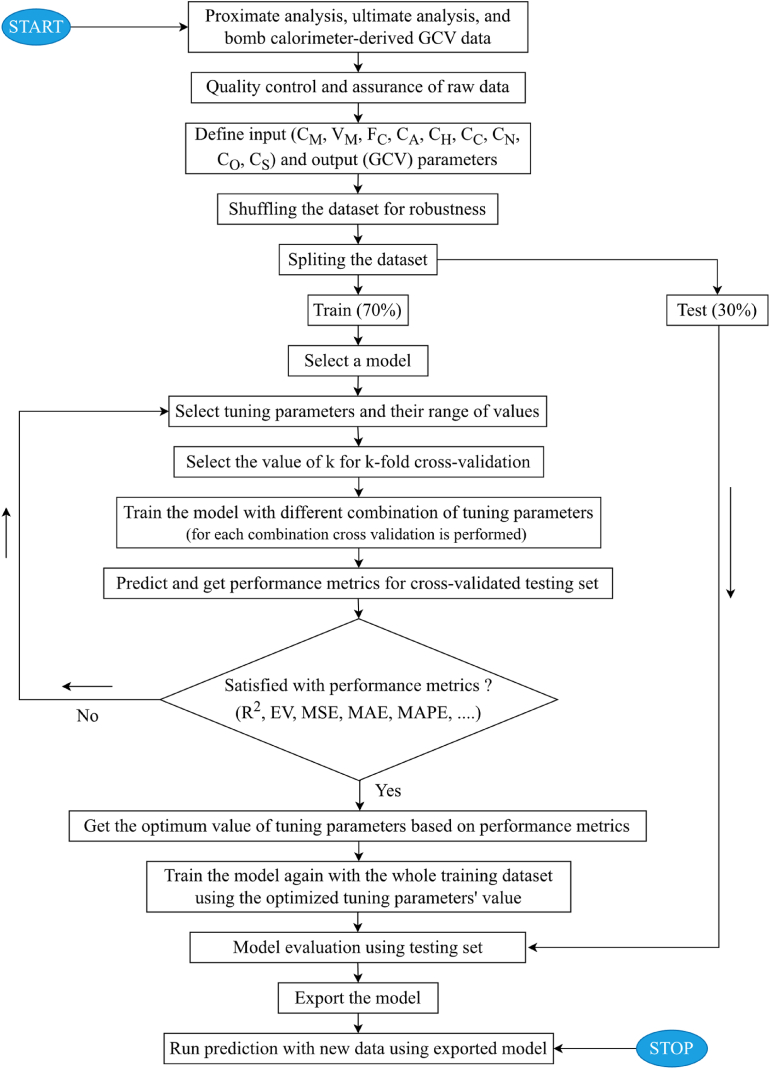
A flow diagram of smart tree-based modeling.

A brief description of the theoretical concept and structure of smart tree-based statistical models is presented in this section. In addition, the methodology explaining the major steps involved in tree-based modeling (Fig. 3) is described.

#### 2.4.1 Decision tree

A decision tree is a binary tree that recursively splits a dataset until pure leaf nodes are left. It has three nodes, i.e., parent nodes, internal nodes, and leaf nodes. A Parent node has no incoming link, internal nodes have both incoming and outgoing links, and leaf nodes only have an incoming link. Various decision tree algorithms, including C4.5, C5.0, ID3, and CART, are employed for various applications. In CART operation, the dataset is split into two sections based on impurity analysis. The splitting procedure is continued until no impurity is left in the dataset. Gini impurity and entropy analysis are performed to measure the impurity, where variance is used as a measure of impurity [49,50]. Binary splitting using a minimum sum of squares is computationally infeasible. Hence, a greedy algorithm is used to perform this binary partition task [51]. For prediction, the predictor space is divided into high-dimensional different-sized boxes, and the values in each box are averaged to get a prediction value for the observation that falls into that region.

#### 2.4.2 Bagging, random forest, and extra trees

Bagging is an ensemble learning method. It employs the same algorithm multiple times to train the model with the same amount of randomly picked and replaced data. After that, the model is trained using all the predictors. The aggregation function (mode or average) combines all the predictors into one. Aggregation lowers the model's variance, a bottleneck in the decision tree method [[51], [52], [53]].

Another ensemble approach that makes use of the bagging technique is random forest. However, unlike Bagging, it uses only decision trees as its base estimator. Another significant difference is that it searches for the best feature from a random selection of features rather than searching for the best feature from the whole feature space while splitting a node. As a result, choosing an arbitrary selection of features for the best feature provides more unpredictability to the model, resulting in a reduced variance in exchange for increased bias [51,54,55].

Random forest is further optimized by introducing more randomness to the bagging ensemble model. When splitting the node, it chooses a random threshold value rather than looking for the best threshold value for each feature. As the threshold value is randomly picked, it reduces computational complexity, which is a strength for this model compared to other bagging ensemble models. It also picks whole training samples instead of bootstrap samples. Due to its severe randomness, this model is called extremely randomized tree ensembles or extra trees for short [56].

#### 2.4.3 Boosting techniques - adaptive boosting, gradient boosting, and XGBoost

Boosting is a popular ensemble technique that turns several weak learners into one strong learner. Unlike Bagging, where trees/predictors are trained parallel and then combined to form a single predictor, Boosting is trained sequentially in an iterative process using data from the previous iteration [52,57]. Numerous boosting algorithms are available, the most popular of which are gradient boosting, adaptive boosting, and extreme gradient boosting (XGBoost).

In adaptive boosting, all the samples are given the same weight at the start, set to the 1/total number of samples, making each sample equally valuable. The first predictor is trained via the training set, and the weighted error rate is determined. The mathematical expressions for weighted error rate (for $j^{\text{th}}$ predictor), predictor weight (for $j^{\text{th}}$ predictor), and weight update rule (for i=1,2,…….,m) are presented in Equation (8), Equation (9), and Equation (10), respectively [55]. The weights of all instances are then standardized. Finally, a new predictor is trained using the revised weights, and the process is repeated.

(8) $$r_{j}=\frac{\sum_{\begin{array}{c} i=1\\ {\hat{y}}_{j}^{\left(i\right)}\neq y^{\left(i\right)} \end{array}}^{m}w^{\left(i\right)}}{\sum_{i=1}^{m}w^{\left(i\right)}}$$

(9) $$\propto_{j}=\eta \;\log\frac{1-r_{j}}{r_{j}}$$

(10) $$w^{\left(i\right)}<-\;\left\{ \begin{array}{c} w^{\left(i\right)}\;\text{if}\;{\hat{y}}_{j}^{\left(i\right)}=y^{\left(i\right)}\\ w^{\left(i\right)}\;\exp\left(\propto_{j}\right)\;\text{if}{\hat{y}}_{j}^{\left(i\right)}\neq y^{\left(i\right)} \end{array}\right.$$

Where ${\hat{y}}_{j}^{\left(i\right)}$ refers to the $j^{\text{th}}$ predictor's prediction for the $i^{\text{th}}$ instance, η denotes the learning rate, $w^{\left(i\right)}$ stands for the weight for $i^{\text{th}}$ instance, $r_{j}$ represents the weighted error rate of the $j^{\text{th}}$ predictor, and $\propto_{j}$ is the predictor's weight.

In contrast to adaptive boosting, which updates its weights at every iteration, in the gradient boosting technique, the prior predictor's residual errors are fit to the new predictor. After the first predictor predicts its value, the pseudo-residual is calculated, and that residual error is used as a label/target for the next iteration using the same inputs/features. The residual error is reduced iteratively by passing the residual error to the next predictor. For the regression task, the first predictor is nothing but the mean value of the target, which is used in the first iteration to calculate residual error [52,58].

Tianqi Chen and Carlos Guestrin of the University of Washington were the ones who first created the XGBoost model. They have built a tree-boosting system (scalable and end-to-end) that incorporates several core innovations. Due to its system and algorithmic optimizations, this model can process data much faster than existing solutions and scales to billions of instances in memory-constrained situations. Tianqi Chen and Carlos Guestrin developed a novel sparsity-aware split finding algorithm that can handle sparsity of data, a theoretically proven weighted quantile sketch for handling weighted data, and a cache-aware block structure for alleviating the slowdown of split finding. These algorithmic and data structure improvements made this model a perfect candidate for a go-to algorithm for a vast and diverse set of datasets [59].

### 2.5 Ranking of proximate and ultimate analyses features

**Fig. 4:**
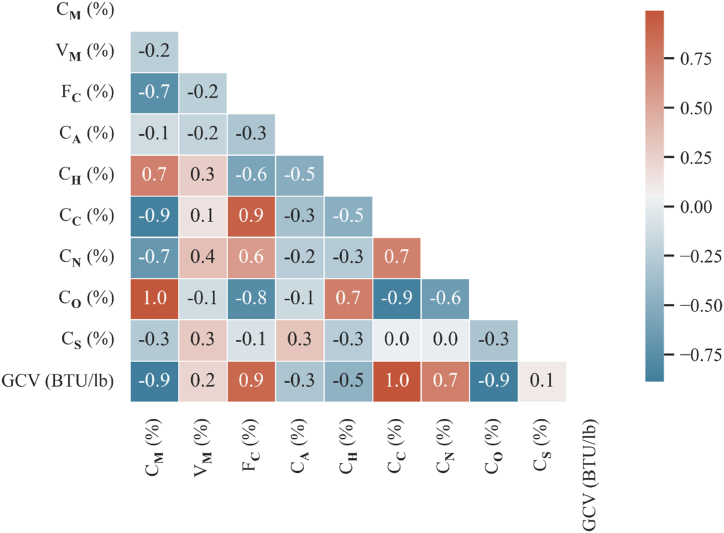
Correlation heatmap for input features and target (GCV).

**Fig. 5:**
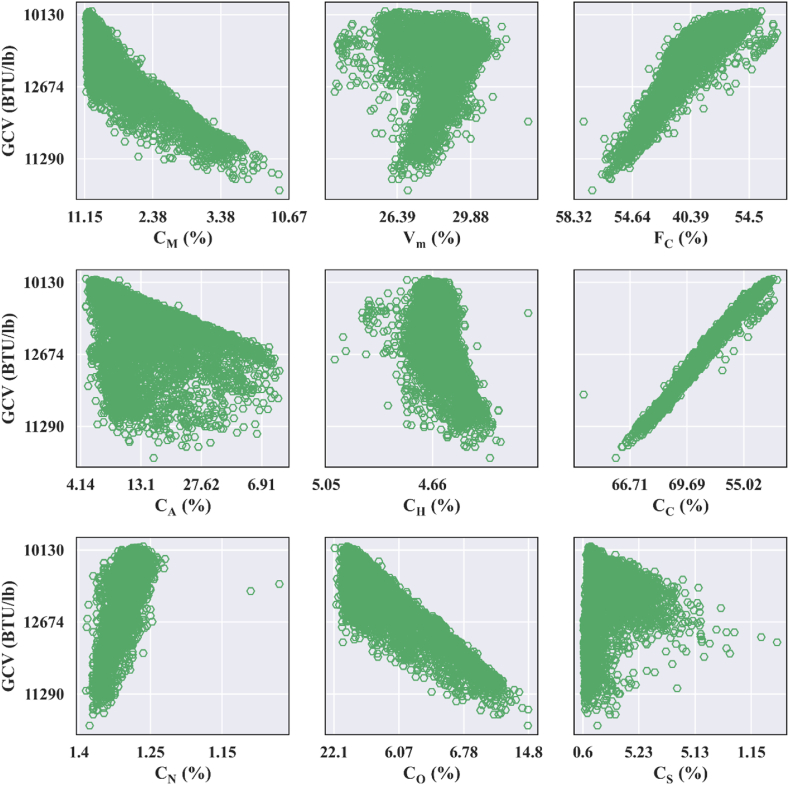
Scatterplots of proximate and ultimate analyses constituents against GCV.

To know the significance of proximate and ultimate analysis features in predicting GCV, the correlation heatmap for input features and GCV (Fig. 4) and scatter pots of ultimate and proximate analyses components versus the target (GCV) (Fig. 5) are developed.

## 3 Results and discussions

### 3.1 Results

#### 3.1.1 GCV estimation using empirical models

All the mathematical relationships reviewed in Section 2.3 presume linear correlation among GCV and ultimate and/or proximate analyses constituents. Scatter plots produced through mapping ultimate and proximate analyses components versus the GCVs (Fig. 5) are used to validate this inherent assumption. Fig. 5 shows a clear linear association between GCV and the variables $C_{M}$, $F_{C}$, $C_{c}$, $C_{N}$, and $C_{O}$. However, considerable scatter among GCV and $V_{m}$, $C_{A}$, $C_{H}$, and $C_{S}$ suggest non-linear dependence between them. These findings imply that the inherent assumption of empirical relationships is incorrect, which suggests that the empirical correlations’ derived GCVs are erroneous. This can also be understood from Table 4. It is evident from Table 4 that the GCV data estimated using empirical models match poorly with actual GCV. The coefficient of determinant is negative, while the error values are substantial for all the evaluated empirical correlation models.

**Table 4:** Statistical performance indexes for empirical models.

Empirical models	$R^{2}$	EV	MSE	MAE	max_error	min_error	MAPE
Schuster [10]	−2	0.001	13810648.2	2901	11606	0	0.33
Spooner [11]	−3	0.024	23170208.6	4218	12834	540	0.45
Mazumdar [12]	−4	0.008	29747260.3	4928	13613	1237	0.51
Mazumdar [13]	−18	−0.969	106403056	9793	14607	20	0.82
Channiwala and Parikh [14]	−24	0.019	135982636	11427	15092	2770	0.99
Parikh et al. [15]	−24	0.014	136540649	11450	15114	2783	0.99
CFRII formulae	−46176228	−124.673	2.5436E+14	15948647	15988584	15834464	1463.96

#### 3.1.2 GCV estimation using smart tree-based models

The values of $R^{2}$ for the testing sets of all the smart tree-based models, such as random forest, extra trees, decision tree, bagging, gradient boosting, adaptive boosting, and XGBoost, are significantly closer to 1, as shown in Table 5**.** The variance of $R^{2}$ for these models is also quite low. Other performance parameters, such as MSE, MAE, and max_error of these models, show a moderate degree of variance, which would be helpful to rank these models. The values of MAE, MSE, min_error, max_error, and MAPE are higher than other smart tree-based models for adaptive boosting. Considering all the performance parameters, XGBoost and extra trees have a presidency over all other models. The hierarchy of the tree-based models based on R^2^, EV, MSE, MAE, MAPE, max_error, and min_error is as follows: XGBoost, extra trees, random forest, bagging, gradient boosting, decision tree, and adaptive boosting. This ranking is performed using plural voting of the statistical indexes.

**Table 5:** Statistical indexes of training and prediction efficiency for smart tree-based models.

Models	$R^{2}$		EV		MSE		MAE		max_error		min_error		MAPE	
	Train	Test	Train	Test	Train	Test	Train	Test	Train	Test	Train	Test	Train	Test
Decision tree	1.00	0.99	1.00	0.99	5119.0	33542.7	49.9	133.3	587.7	1417.0	0.000	0.000	0.004	0.013
Bagging	1.00	1.00	1.00	1.00	3400.2	20985.1	37.9	101.7	977.3	1604.4	0.025	0.000	0.004	0.010
Random forest	1.00	1.00	1.00	1.00	3871.9	20376.9	38.3	100.5	1361.2	1593.2	0.002	0.027	0.004	0.010
Extra trees	1.00	1.00	1.00	1.00	0.0	16030.6	0.0	91.3	0.0	1357.0	0.000	0.000	0.000	0.009
Adaptive boosting	0.98	0.98	0.98	0.98	108204.9	113781.0	264.0	269.0	1071.4	1641.3	0.082	0.286	0.024	0.025
Gradient boosting	1.00	1.00	1.00	1.00	15016.0	22485.0	88.7	111.8	2688.6	1124.1	0.028	0.039	0.008	0.011
XGBoost	1.00	1.00	1.00	1.00	8234.4	14703.3	68.2	89.2	1184.4	1027.2	0.026	0.116	0.006	0.009

The optimum hyperparameter values used in smart tree-based models are shown in Table 6, along with their grid-search run time. Although the combinations of tunning parameters of XGBoost are higher than random forest and extra trees, XGBoost takes less time to run all those combinations. Hence, its algorithmic optimization of data handling is quite visible here.

**Table 6:** Hyperparameter tuning results of smart tree-based models.

Model	Tuning parameter	Range of values	Optimal value of tuning parameter	GridSearch run time (h:m:s)
Decision tree	max_depth	Range (1:30)	11	0:00:06
Bagging	n_estimators	20, 30, 40, 50, 60, 70, 80	40	0:01:15
Random forest	n_estimators	50, 70, 90, 100, 110, 130, 150	100	2:17:47
	criterion	squared_error, friedman_mse, absolute_error, poisson	friedman_mse	
	max_depth	1, 2, 3, None	None	
	min_samples_split	2, 3, 4, 5	3	
Extra trees	n_estimators	10, 50, 100, 150, 200, 300	300	1:34:59
	criterion	squared_error, absolute_error, friedman_mse, poisson	friedman_mse	
	max_depth	1, 2, 3, None	None	
	min_samples_split	2, 3, 4, 5	2	
Adaptive boosting	n_estimators	70, 75, 80, 90, 100, 110, 120	100	0:04:13
	learning_rate	0.1, 0.3, 0.5, 0.7, 0.9, 1	0.9	
	loss	linear, square, exponential	square	
Gradient boosting	loss	squared_error, absolute_error, huber, quantile	huber	0:38:49
	learning_rate	0.1, 0.3, 0.5, 0.7, 0.9	0.3	
	n_estimators	10, 20, 30, 40, 50	50	
	max_depth	1, 2, 3, None	3	
XGBoost	n_estimators	100, 300, 400, 500, 600, 700, 800	500	0:44:43
	max_depth	1, 2, 3, None	3	
	grow_policy	depthwise, lossguide	depthwise	
	learning_rate	0.001, 0.05, 0.01, 0.1	0.1	
	early_stopping_rounds	3, 5, 7	7	
	tree_method	exact, approx, hist	exact	
**Specification of the working computer**				
Processor	AMD Ryzen 7 5700G with Radeon Graphics		L1 cache	512 KB
Base clock speed	3.8 GHz		L2 cache	4.0 MB
Cores	8		L2 cache	16 MB
RAM	16 GB			

#### 3.1.3 Input variables (proximate and ultimate analyses features) ranking

Fig. 4 (inter-item correlation heatmap) and Fig. 5 (scatter plots) represent the dependence of GCV on proximate and ultimate analysis features. Fig. 4, Fig. 5 imply that the $C_{C}$ and $F_{C}$ display a highly positive correlation coefficient with GCV, whereas $C_{M}$ and $C_{O}$ show worthwhile negative correlation. Therefore, it can be recommended that the $C_{C}$, $F_{C}$, $C_{M}$, and $C_{O}$ are highly influential input parameters for GCV. The $C_{O}$, $C_{C}$, and $C_{M}$ are the most critical rank parameters for GCV prediction, whereas $C_{A}$, $V_{m}$, and $C_{S}$ are the least significant variables.

### 3.2 Discussions

The present study develops intelligent statistical models, namely Extra trees, Bagging, Decision tree, and Adaptive boosting for the first time in coal's GCV modeling to increase/improve the estimation accuracy. Prediction and computation efficiency-based comprehensive comparison among well-established empirical models and newly developed bagging and boosting algorithms is also a novelty of this research. Another scientific originality of this study is the ranking of ultimate and proximate analyses parameters based on their significance in GCV modeling.

Well-established empirical models, such as Schuster [10], Mazumdar [12], Mazumdar [13], Channiwala and Parikh [14], Parikh et al. [15], and CFRII formulae provide negative values of $R^{2}$. According to the definition of $R^{2}$ given in Equation (1), when the ratio of $SS_{\text{res}}$ and $SS_{\text{tot}}$ is greater than 1, the value of $R^{2}$ could be negative. It happens when the model fits the original data very poorly. Hence, the negative $R^{2}$ value obtained from the studied empirical models indicates that these models are arbitrarily worse. Obtained erroneous GCVs suggest that the studies of coal mines are incompatible with the inherent assumptions and foundation of these empirical models. In general, empirical models cannot capture the non-linear correlations between GCV and ultimate and/or proximate constituents. Hence, empirical modeling is inappropriate for predicting GCV.

**Fig. 6:**
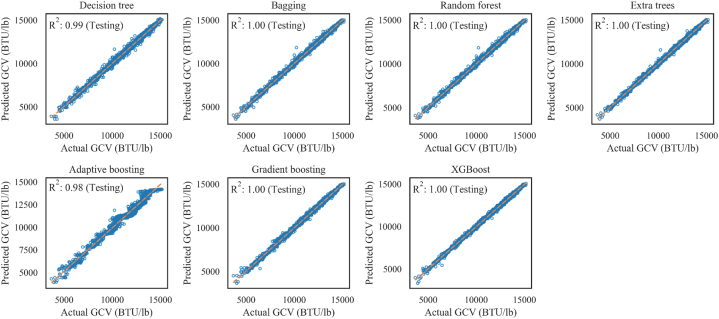
Correlation of original and predicted GCV of decision tree, bagging, and boosting techniques (Testing data set).

The smart tree-based models employed here perform efficiently as they all provide quite good performance. The $R^{2}$ values for all these models are over 0.97. However, the XGBoost model outperforms other smart tree-based models with the most significant coefficient of determinant ($R^{2}$ of 0.9974) and lowest error values (MSE of 14703.3, max_error of 1027.2, MAE of 89.2, MAPE of 0.009). As XGBoost and extra trees predicted GCVs perfectly fit with actual GCVs (Fig. 6), they could be proposed as a suitable GCV predicting strategy.

All studied empirical models provide very low $R^{2}$ and very high error measures. Hence, well-established empirical algorithms are inappropriate for GCV modeling. The smart tree-based models are highly capable of predicting GCV as their correlation coefficient and involved error are high and low, respectively.

The feature ranking reveals that the $C_{O}$, $C_{C}$, and $C_{M}$ are the most significant input parameters for GCV prediction, whereas $C_{A}$, $V_{m}$, and $C_{S}$ are the least important variables. The variable ranking can assist operators in selecting a few features rather than conducting comprehensive coal analysis to predict GCV, consequently saving the tedious and time-consuming laboratory effort.

The strategy suggested in this research can aid engineers/operators in obtaining a rapid and accurate determination of the GCV with a few coal features, thus lessening laboratory efforts and significantly lowering the experimental costs. In addition, the developed decision tree-based models can assist geochemists/engineers in precisely evaluating coal's practical energy matter and accurately characterizing coal mines. This study will likely open an entrance for introducing decision tree-based bagging and boosting techniques in fuel engineering studies where innovative connectionist models will be applied instead of well-established empirical algorithms to recognize inherent highly complex links from input-output data, find optimum patterns, and forecast target parameters.

## 4 Conclusions

i) The existing empirical models applied to derive coal's GCV from ultimate and proximate analyses are linear in nature. It is seen that there is a clear linear relationship among GCV and $C_{M}$, $F_{C}$, $C_{c}$, $C_{N}$, and $C_{O}$. However, considerable non-linear dependence of GCV on $V_{m}$, $C_{A}$, $C_{H}$, and $C_{S}$ is also observed. Hence, empirical modeling is inappropriate for predicting GCV. The prediction performance of all empirical models is abysmal and erroneous.
ii) The studied tree-based models are not very distinct from each other. The $R^{2}$ values for these models are very high, suggesting an excellent match between actual and predicted GCVs.
iii) The comprehensive contrast among studied methods demonstrates that the hierarchy of the applied models based on statistical measures and computational efficiency is as follows: XGBoost, Extra trees, Random forest, Bagging, Gradient boosting, Decision tree, and Adaptive boosting, Schuster [10], Spooner [11], Mazumdar [12], Mazumdar [13], Channiwala and Parikh [14], Parikh et al. [15], CFRII formulae.
iv) The $C_{O}$, $C_{C}$, and $C_{M}$ are the most crucial input variables for GCV prediction, whereas $C_{A}$, $V_{m}$, and $C_{S}$ are the least important variables.

## Data availability statement

Data will be made available on request.

## CRediT authorship contribution statement

**Tanveer Alam Munshi:** Writing – original draft, Software. **Labiba Nusrat Jahan:** Writing – review & editing, Writing – original draft, Project administration. **M. Farhad Howladar:** Writing – review & editing, Writing – original draft. **Mahamudul Hashan:** Writing – review & editing, Writing – original draft, Supervision, Project administration, Conceptualization.

## Declaration of competing interest

The authors declare that they have no known competing financial interests or personal relationships that could have appeared to influence the work reported in this paper.

## Abbreviations

AAPD: Absolute average percent deviation
ACE: Alternating conditional expectation
ANFIS: Adaptive neuro fuzzy inference system
ANN: Artificial neural network
APD: Average percent deviation
ASTM: American Society for Testing and Materials
BPNN: Backpropagation neural network
CANFIS: Co-active neuro-fuzzy inference system
CART: Classification and regression tree
CCV: Coal calorific value
CFRII: Central Fuel Research Institute of India
CNN: Convolutional neural network
EV: Explained variance
FDT: Fuzzy decision tree
FL: Fuzzy logic
GA: Genetic algorithm
GBT: Gradient boosting trees
GCV: Gross calorific value
GMDH: Group method data handling
GRNN: General regression neural network
ICA: Imperialist competitive algorithm
MAE: Mean absolute error
MAPE: Mean absolute percentage

**Error**
ME: Mean error
MSE: Mean squared error
MLP: Multilayer perceptron
MLR: Multiple linear regression
MVR: Multiple variable regression
PSO: Particle swarm optimization
RBF: Radial basis function
RF: Random forest
RNN: Recurrent neural network
SD: Standard deviation
SHAP: Shapley additive explanations
SVM: Support vector machine
SVR: Support vector regression
TOB: Transparent open-box
UHV: Useful heating value
VAF: Variance
WNN: Wavelet neural network

**Variables, parameters, symbols, subscripts, and Greek letters**
$C_{A}$: Concentration of ash (%)
$C_{C}$: Concentration of carbon (%)
$C_{H}$: Concentration of hydrogen (%)
$C_{M}$: Concentration of moisture (%)
$C_{N}$: Concentration of nitrogen (%)
$C_{O}$: Concentration of oxygen (%)
$C_{S}$: Concentration of sulfur (%)
$F_{C}$: Concentration of fixed carbon (%)
Q: Gross calorific value
R_max_: Vitrinite maximum reflectance
R: Regression coefficient
$R^{2}$: Coefficient of determinant
SS_res_: Sum of squares of residuals
SS_tot_: Total sum of squares
$V_{m}$: Concentration of volatile matter (%)
y: True output value
$\hat{y}$: Predicted output value
$\bar{y}$: Mean of y
$\hat{y_{i}}$: Predicted output value of the i-th sample
$y_{i}$: True output value of the i-th sample
${\hat{y}}_{j}^{\left(i\right)}$: Predicted value of the $j^{\text{th}}$ predictor for the $i^{\text{th}}$ instance
$\propto_{j}$: Predictor's weight
η: Learning rate
$r_{j}$: Weighted error rate of the $j^{\text{th}}$ predictor
ϵ: Small positive constant
$w^{\left(i\right)}$: Weight for $i^{\text{th}}$ instance
